# Update in autoimmune and paraneoplastic myelopathies: Newly described antigen targets and antibody testing

**DOI:** 10.3389/fneur.2022.972143

**Published:** 2022-07-28

**Authors:** Michlene Passeri, Elizabeth Matthews, Ryan Kammeyer, Amanda L. Piquet

**Affiliations:** ^1^Department of Neurology, University of Colorado Anschutz Medical Campus, Aurora, CO, United States; ^2^Department of Pediatrics and Neurology, Children's Hospital Anschutz Medical Campus, Aurora, CO, United States

**Keywords:** myelopathy, myelitis, autoimmune manifestations, NMO spectrum disorder (NMOSD), MOG antibody associated diseases, paraneoplastic syndrome (PNS)

## Abstract

Myelopathy is an increasingly recognized presentation of many antibody-mediated neuroinflammatory disorders. While specific features of certain autoimmune myelopathies such as aquaporin-4 antibody associated neuromyelitis optica spectrum disorder (NMOSD) and myelin oligodendrocyte glycoprotein associated disorder (MOGAD) are well-characterized, other less commonly seen antibody-associated myelopathies are not as well-defined. These include but are not limited to, Hu/ANNA1, anti-glial fibrillary acidic protein (GFAP), anti-CV2/collapsin response mediator protein (CRMP5), and amphiphysin. Here, we review the mentioned more common antibody mediated myelopathies as well those that as less common, followed by a review of differentials that may mimic these disorders.

## Introduction

Autoimmune and paraneoplastic myelopathies are a heterogenous group of disorders. Early and accurate diagnoses of these conditions improves clinical outcomes (1). Some autoantibodies in this group of disorders are considered pathogenic such as aquaporin-4 (AQP4) in neuromyelitis optica spectrum disorder (NMOSD), whereas other antibodies are not directly pathogenic but are rather markers of cytotoxic T cell mediated autoimmunity [i.e., collapsin receptor mediator protein-5 (CRMP5)]. These indirect pathogenic mechanisms occur in syndromes with an intracellular antigen target as opposed to antibodies against cell surface antigen targets. Clinical history, unique radiographic findings, and identification of autoantibodies can help guide appropriate treatment. In this review, we describe autoimmune myelopathies such as AQP4-IgG positive NMOSD and anti-myelin oligodendrocyte glycoprotein (MOG) antibody associated disease (MOGAD), in addition to less commonly described antibodies including anti-CRMP5, anti-glial fibrillary acidic protein (GFAP), amphiphysin, Hu/ANNA1, among others.

## Methods

We performed a systematic literature search in PubMed to identify autoantibodies reported against neuronal targets in autoimmune myelopathies. To help guide clinicians, we also included antibody syndromes that recently became commercially available for testing in the United States as of 2022 (2).

### Anti-aquaporin-4 antibody

NMOSD is an antibody-mediated disease of the central nervous system associated with AQP4-IgG (1, 3). There are three cardinal features: transverse myelitis, optic neuritis, and area-postrema syndrome (4). Most experience a relapsing course, and relapses are often severe and associated with disability. NMOSD is 5–10 times more common in women, with a median age of 40 (1, 3).

AQP-4 is located on the end feet of astrocytes. Binding of AQP4-IgG leads to an inflammatory cascade, ultimately resulting in secondary demyelination. AQP4-IgG is highly specific and highly sensitive for NMOSD. Optimal testing uses a blood cell-based assay (CBA) with fluorescence-activated cells sorting (FACS) (5).

**Figure 1:**
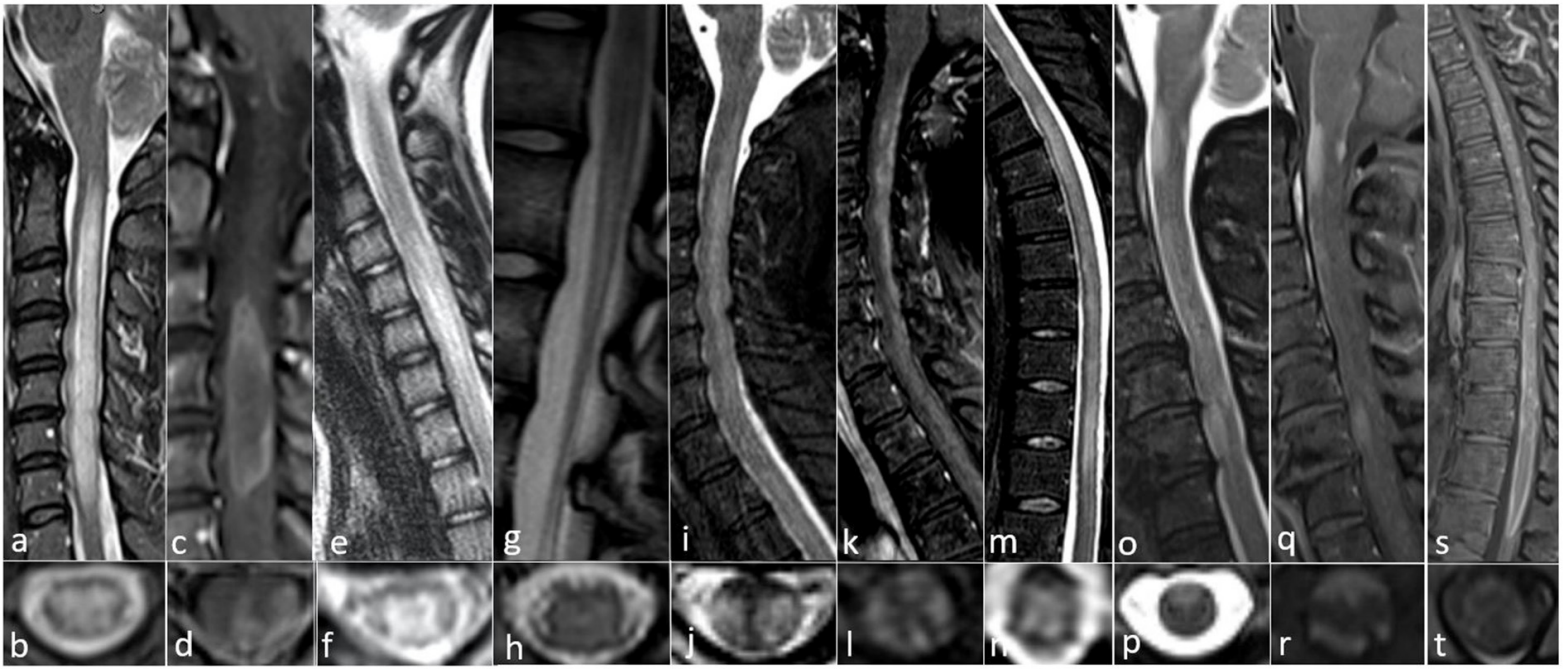
All images collected from cases seen at the Autoimmune and Neuroimmunology clinics at the University of Colorado. **(a–d)** AQP4-IgG positive 52-year-old woman with progressive limb weakness and sensory loss with MRI showing **(a)** sagittal confluent T2 hyperintensity with cord edema from C2 to T1, **(b)** axial central predominant T2 hyperintense lesion, **(c)** sagittal ring-enhancement pattern from C3 to C5, and **(d)** Axial ring enhancement pattern with central and lateral involvement. **(e–h)** MOG IgG positive 12-year-old-girl with gait abnormality and mild lower extremity weakness with MRI showing **(e)** sagittal T2 hyperintensity with mild cord edema from C4 to C7, **(f)** axial gray matter T2 hyperintensity with appearance of an “H” sign, **(g)** sagittal T2 hyperintensity of the conus medullaris, and **(h)** Axial subtle symmetric central T2 hyperintensity. **(i–n)** CRMP5 IgG positive 54-year-old woman with paraplegia, found to have a neuroendocrine carcinoma suggestive of thymus or small cell lung origin and MRI with **(i)** sagittal T2 hyperintensity with cord swelling from C2 to T1, **(j)** axial posterior and lateral T2 hyperintensity, **(k)** sagittal dorsal subpial contrast enhancement from C2 through T1, **(l)** axial anterior, lateral, and dorsal subpial contrast enhancement, **(m)** sagittal T2 hyperintensity from T1 to T11, and **(n)** axial central predominant T2 hyperintensity. **(o–t)** GFAP IgG positive 63 year old woman with subacute progressive encephalomyelitis with no underlying malignancy and MRI findings of **(o)** sagittal patchy, extensive ventral T2 hyperintense lesions from the craniocervical junction to C7, **(p)** Axial ventral T2 hyperintensity, **(q)** sagittal dorsal and central enhancement at the margin of the craniocervical junction as well as ventral enhancement at the margin of the cord at C3 and C6, **(r)** axial dorsal and ventral subpial enhancement, **(s)** sagittal diffuse enhancement of the ventral and dorsal margins of the distal thoracic cord from T8 to T11, and **(t)** axial ventral subpial enhancement.

Longitudinally extensive myelitis (LETM), defined as a myelitis extending ≥3 or more continuous vertebral segments, is commonly seen and frequently recurrent in NMOSD (1). Short segment lesions can occur in 15% of NMOSD attacks, which can be mistaken as multiple sclerosis (MS) (6). Lesions are often located centrally sometimes with associated ring enhancement (3) (Figures 1a–d).

Coexisting systemic autoimmunity is common, including systemic lupus erythematosus, Sjögren syndrome, and antiphospholipid syndrome. Given the high specificity of AQP4-IgG, the neurologic manifestations are expected to be secondary to AQP4-IgG as opposed to the rheumatologic condition. Myasthenia gravis can also coexists with NMOSD (3).

### Anti-myelin oligodendrocyte glycoprotein

MOGAD is characterized by demyelinating attacks often with syndromes of optic neuritis, transverse myelitis, AQP4-IgG negative NMOSD, acute disseminated encephalomyelitis (ADEM), and brainstem demyelinating episodes (7). MOGAD may be multiphasic (more common in adults) or monophasic (more common in children) especially with ADEM presentations (8). There is slight female predominance (1.5:1, Female:Male) (7).

MOG is a protein located on the outer membrane of myelin sheaths that is produced by oligodendrocytes and plays a role in the formation and function of the myelin sheath. While its function is not fully elucidated, proposed mechanisms include cell adhesion, regulating microtubule stability and modulating myelin immune interactions (9). Optimal testing is serum CBA and is only recommended when a patient has a syndrome commonly seen in MOGAD due to the risk of false positivity (10, 11). A higher MOG-IgG titer (i.e., > 1:100), portends a higher degree of diagnostic certainty, as the positive predictive value tends to vary with autoantibody titer (≥1:1,000, 100%; 1:100, 82%; 1:20–40, 51%) (10).

Myelitis lesions in MOGAD are longitudinally extensive in 60–80% of cases, can involve the conus medullaris and are typically more severe in clinical presentation compared to MS, but usually have better recovery than AQP4-IgG positive NMOSD. Recurrent LETM is rare (around 2%) whereas this is common with AQP4-IgG (up to 90% of cases).

On MRI, central gray matter T2 hyperintensities are seen in one third of cases appearing as an “H” sign on axial view (Figures 1e–h). This is opposed to AQP4-IgG positive cases which typically involve both white and gray matter (3). MRI features also include non-enhancing lesions. Compared to AQP4-IgG NMOSD, spinal lesions resolved more frequently in MOGAD without chronic spinal atrophy which can be seen in AQP4-IgG myelitis (12). MRI-negative myelitis can be seen in patients with acute/subacute myelopathic syndromes in the setting of positive MOG-IgG (13).

### Anti-glial fibrillary acidic protein

Anti-GFAP astrocytopathy commonly present as an immunotherapy-responsive meningoencephalomyelitis. Cancer is seen in about 34% of cases, with ovarian teratoma being the most common, often with the coexistence of N-methyl-D-aspartate receptor (NMDAR) antibodies (14).

The hallmark radiologic feature is periventricular, radial, linear enhancement on brain MRI, and testing is most specific in the cerebrospinal fluid (CSF) *via* CBA. GFAP is a cytosolic intermediate filament protein of astrocytes. It is thought to not be directly pathogenic, but rather a biomarker of inflammation (15). Spinal fluid analysis typically shows pleocytosis (88%), elevated protein (83%), CSF-exclusive oligoclonal bands (54%), and hypoglycorrhachia (18%) (14).

Myelitis has been demonstrated in 30% of patients (16), with one study finding myelitis as the presenting symptom in 10.5% (14). Isolated myelitis is rare and in onset study identified in 5% (17). Spinal cord lesions are often longitudinally extensive with an enhancement pattern that is thin, linear, and patchy, involving the central canal or leptomeninges (14). Typically the T2 signal changes seen in anti-GFAP astrocytopathy tend to be more subtle and hazy compared to AQP4-IgG or MOG-IgG related LETM. There can be prominent enhancement of the central cord since there are GFAP-enriched regions adjacent to the central canal (17) (Figures 1o–t).

Patients may have a monophasic, relapsing, or progressive course despite treatment (14). Other presenting symptoms outside of myelitis may include rhomboencephalitis, cerebellar dysfunction, swallowing difficulties, cognitive difficulties, seizures, parkinsonism, myoclonus, dysautonomia, optic disc edema, optic neuritis, uveitis, and cranial nerve palsies affecting vision (14, 16).

Coexisting autoimmunity, including AQP4, MOG, and NMDAR antibodies have been reported (14, 16). T cell dysfunction is common, and cases of HIV, lymphopenia, exposures to anti-programmed cell death inhibitors (18), and anti-tumor necrosis factor inhibitors have been reported (16).

### Anti-collapsin response-mediator protein-5

CRMP5 and amphiphysin are the two most common paraneoplastic antibodies associated with myelopathy (19). CRMP5-associated paraneoplastic neurologic syndrome (PNS), originally named anti-CV-2 (20), commonly presents with painful neuropathy and/or cerebellar ataxia with a strong association with small cell lung cancer (SCLC) (20). Myelopathy, described in 19% of 105 patients, was the third most common presentation. Similarly, myelopathy was present in 16% of cases in another review (1, 21). In addition to SCLC, thymomas may be seen, and cancer is found in about 73% of cases (22).

Myelopathy has been characterized as monophasic in 18%, relapsing in 15%, and progressive in 68% which has been mistaken for MS (23). Myelopathic patterns described in a cohort of 57 cases include LETM in 42%, with gadolinium enhancement in 43% (23) (Figures 1i–n). Cauda equina enhancement has also been reported (21). CSF is typically inflammatory, with an elevated protein or nucleated cell count in 92% (21). Co-existing paraneoplastic antibodies have been reported, most commonly anti-Hu (22). High dose steroids have shown significant benefit with pain and neuropathic symptoms (21). In a cohort of 57 CRMP5-associated myelopathy, 12 of 16 patients (75%) stabilized or improved with corticosteroids and/or immunosuppressive therapy or tumor eradication in three cases (23).

### Anti-amphiphysin

Anti-amphiphysin antibodies are seen in PNS most commonly associated with breast cancer (~90% of cases) as well as ovarian and SCLC (24, 25). This antibody was first described in a rare form of paraneoplastic stiff person syndrome (SPS), a disorder characterized by symmetric muscle stiffness and painful spasms of axial and limb muscles, without pyramidal or extrapyramidal signs (26). They do not present with “classic” SPS given other associated neurologic symptoms (26). It is now recognized that anti-amphiphysin is associated with a broad range of neurologic presentations including SPS, polyradiculoneuropathy, diffuse sensory neuronopathy, encephalomyelitis, limbic encephalitis, Lambert Eaton myasthenic syndrome (LEMS), cerebellar degeneration, opsoclonus-myoclonus, as well as myelopathy (24, 27, 28).

Reported cases of myelopathy are typically LETM including: lateral tract specific LETM from C3 to T12 (19), a case of coexisting Hu and amphiphysin antibodies with symmetric, bilateral, tract specific (dorsal column) LETM with concomitant length-dependent sensory polyneuropathy (29), a report of LETM from C2-C7 associated with breast cancer (30), and a case of symmetric involvement of the lateral tracts from T2 through T7 in association of breast adenocarcinoma (31). Outcomes are variable, however are more favorable to immunotherapy with isolated amphiphysin antibodies (28).

### Anti-neuronal nuclear antibody-1

ANNA-1, or anti-Hu, is a well-characterized PNS, associated with SCLC in 74–94% of cases (32). While SCLC is most common, a number of other cancers have been associated including breast, prostate, gastrointestinal, ovarian and bladder cancer (32, 33). Anti-Hu antibodies target an intracellular antigen and are therefore likely a marker of a broader cytotoxic T cell-mediated immune response as opposed to directly pathogenic.

Myelitis is an uncommon manifestation, seen in roughly 11% of cases (1, 32, 33), with other clinical presentations seen including sensory neuropathy, limbic encephalitis, brainstem encephalitis, cerebellar ataxia, chronic intestinal pseudo-obstruction, and dysautonomia with most patients presenting with multifocal neurologic manifestations (32). When myelitis does occur, it typically does so in combination with other manifestations of anti-Hu, e.g., encephalomyelitis (32, 33). Tract-specific LETM has been reported with anti-Hu both in isolation and in association with anti-amphiphysin antibodies, both with dorsal column-specific involvement in conjunction with sensory neuronopathy or sensory-predominant axonal polyneuropathy (29).

Outcomes are dependent on timing of diagnosis, disability level at the time of diagnosis, and success of treatment of underlying malignancy, without clear evidence that immunotherapy impacts the overall outcome. Overall survival rates are poor, with 3-year survival rate in one series being 22% (34, 35).

### Other less common autoantibodies associated with autoimmune myelopathy: Anti-DPPX, anti-GAD65, anti-PCA-1, anti-ANNA-2, and others

#### Anti-dipeptidyl-peptidase-like protein 6 (DPPX) antibody

Anti-DPPX is a cell-surface antibody typically seen in syndromes with features of central nervous system hyperexcitability (e.g., psychiatric symptoms, seizures, tremors, hyperkplexia, myoclonus), memory loss and significant weight loss with or without diarrhea (36–39). Additional features include movement disorders, sleep disorders resembling REM-sleep behavior disorder, dysautonomia, cerebellar ataxia, and brainstem and spinal cord disorders. In a review of 53 patients, brainstem or spinal cord disorders were seen in 75.5% of cases, with symptoms including eye movement abnormalities, dysarthria, dysphagia, stiffness, respiratory failure, vertigo, and hyperekplexia (36). In another cohort of 20 patients, 15 had brainstem or spinal cord disorders (39).

DPPX is a regulatory subunit of the voltage-gated A-type (rapidly inactivating) Kv4.2 potassium channel complex expressed in neuronal dendrites and soma (39). DPPX proteins are distributed in the nervous system and enteric system which accounts for gastrointestinal symptoms and weight loss. Serum and CSF seem to be equally sensitive for DPPX-IgG testing (39).

DPPX antibody-associated disease is often immunotherapy responsive (40). Malignancies are less common and include most often B cell neoplasms and rarely breast adenocarcinoma, micropapillary carcinoma of the thyroid (36).

#### Anti-glutamic acid decarboxylase-65

GAD65 autoimmunity is commonly associated with type 1 diabetes, autoimmune thyroid disease, and pernicious anemia. Neurologic autoimmunity may be seen, often in addition to these systemic autoimmune diseases (41). GAD65 autoantibodies are located intracytoplasmically in presynaptic nerve terminals. Like other cytoplasmic or nuclear antibodies, GAD65 antibodies are likely markers of a cytotoxic T-cell mediated process (41). The most common neurologic manifestations are limbic encephalitis, autoimmune epilepsy, cerebellar ataxia and SPS (41). In one large series of 212 patients with GAD65-associated neurologic autoimmunity, 23 patients (11%) were diagnosed with myelopathy based on clinical manifestations including upper motor neuron signs, pyramidal weakness, bowel/bladder dysfunction. None had T2 hyperintensities or gadolinium enhancement of the spinal cord on imaging. The lack of radiographic correlate was similar for those who had clinical manifestations of brainstem dysfunction. None of the cases of myelopathy occurred in isolation (42).

It is worth noting that GAD65 positivity is seen in around 8% in the general population, and does not always indicate an autoimmune disease, particularly at low titers. Patients with neurologic disease typically have markedly elevated titers (>20 nmol/L), whereas patients with type 1 diabetes titers will be less (41). Patients with neurologic disease often have detectable antibodies in the CSF, though typically at lower titers than in serum (41).

#### Anti-purkinje cell cytoplasmic autoantibody type 1/anti-Yo

PCA-1/Anti-Yo antibody, first described in 1983, commonly presents as a subacute, severe pan-cerebellar syndrome associated with breast or gynecologic cancer (43). The neurologic syndrome often precedes the cancer diagnosis. Anti-Yo antibodies are directed against CDR proteins which are primarily expressed on cerebellar Purkinje cells. However, there have been reports of other neurologic syndromes including myelopathy (44). One case report of a 36-year-old woman with breast cancer and progressive lower extremity sensory loss revealed a longitudinally-extensive, dorsal-predominant T2 hyperintensity from T5 to T10 (45). Another case report with available neuropathology demonstrated inflammatory infiltrates and demyelination of the spinal cord tissue (46). These reports suggest more widespread distribution of the Yo proteins outside the Purkinje cells. Outcomes are often poor (44).

#### Anti-neuronal nuclear antibody-2/anti-Ri

ANNA-2/Anti-Ri is associated with cancer in as many as 86% of cases, commonly lung or breast (47). Myelopathy associated with anti-Ri neurologic syndromes are rare. In a review of 34 patients with anti-Ri associated neurologic syndromes, only one had myelopathy. More common syndromes include brainstem syndromes (e.g., opsoclonus-myoclonus), cerebellar syndromes, peripheral neuropathy, movement disorders including dystonia, encephalopathy, seizures, and LEMS (47). There is one case of a woman with breast cancer and progressive spastic quadriplegia with a myelitis involving the conus to the cervicomedullary junction with no response to immunotherapy (48).

#### Others

Myelopathy has been reported with several additional antibodies including metabotropic glutamate receptor 1 (mGluR1), PCA2, and SRY-Box transcription factor 1 (SOX1) but limited to isolated case reports. While myelitis may be a rare manifestation, another potential explanation is these patients may have harbored co-existing antibodies that were not identified (49–51).

## Discussion

### Differential diagnosis

When considering the differential diagnosis, there are a wide variety of etiologies that may mimic autoimmune or paraneoplastic myelopathy including infections, MS, other inflammatory conditions, spinal cord compression, vascular etiologies, metabolic myelopathies and malignancy (Table 1) (1, 52–56).

**Table 1:** Differential diagnosis of myelopathies by etiologic classification and associated clinical and radiographical findings.

**Diagnosis**	**Clinical clues**	**Spinal cord MRI findings**
**Inflammatory/infectious**		
Multiple sclerosis (MS)	Subacute onset Other features of MS (i.e., relapsing- remitting course, other demyelinating events)	Short, peripheral lesions May see ring enhancement
Neurosarcoidosis	Steroid responsive May have systemic granulomatous disease (e.g., pulmonary involvement)	Dorsal subpial enhancement or “Trident sign”, or patchy enhancement with “string-of-pearls” Often longitudinally extensive lesions with radiographical disease out of proportion to clinical disability
Systemic Autoimmune Disease	Known rheumatologic disorder (i.e., lupus or Sjogren's) and other systemic symptoms: rash, sicca syndrome, Raynaud's, etc.	Variable LETM - must rule out comorbid AQP4-IgG positive NMOSD as this can be comorbid with lupus
Infectious Myelopathy	Systemic signs of infection Recent travel or exposures (e.g., tick bite) Immunocompromised Poliomyelitis Myeloradiculitis	Variable Anterior horn involvement (e.g., poliomyelitis particularly with enterovirus)
**Antibody-associated**		
NMOSD	Associated with AQP4-IgG (best tested in serum), Cardinal features of NMOSD include transverse myelitis, optic neuritis, and area-postrema syndrome Often a relapsing course, with attacks severe and associated with disability	Usually a LETM, although short segment myelitis can be seen Lesions located centrally and associated with enhancement in acute attacks (can see ring-enhancement)
MOGAD	Associated with MOG-IgG (best tested in serum) Demyelinating attacks with syndromes of optic neuritis, transverse myelitis, AQP4-IgG negative NMOSD, and acute disseminated encephalomyelitis (ADEM)	Long or short T2 lesions, non-enhancing lesions, conus medullaris involvement, gray matter involvement (“H” sign)
GFAP astrocytopathy	Associated with GFAP-IgG (best tested in CSF), Presents as an immunotherapy-responsive meningoencephalomyelitis Isolated myelitis is rare (5%)	Brain MRI with hallmark features of periventricular radial, linear enhancement. When spinal cord is involved, often LETM with more subtle, hazy T2 signal changes and central cord enhancement.
**Structural**		
Cervical spondylosis	Acute or chronic onset Trauma/hyperextension injury Radiculopathy Central cord syndrome	Lesion at level of disc or structural abnormality Long or short Spares gray matter on axial “Pancake-like” on sagittal
**Vascular**		
Spinal cord infarction	Hyperacute onset Recent vascular (aortic) surgery) Severe pain at onset Trauma (even minor)	“Owl eye” appearance on axial Hyperintense on diffusion weighted imaging Adjacent artery dissection Vertebral body infarct
Spinal dural arteriovenous fistula	Stepwise worsening thoracic myelopathy Worsening with Valsalva or exertion	Lower thoracic cord Patchy enhancement and dilated peri-medullary veins/flow voids
**Toxic**		
Chemotherapy toxicity	Chemotherapy use (most common methotrexate or cytarabine) Rapid onset Incomplete recovery	Long lesion Dorsal and/or lateral T2 hyperintensity
Radiation toxicity	Delayed onset (6–24 months after radiation) Steroid responsive	T2 hyperintense lesion at site of radiation May have contrast enhancement acutely with Chronic cord atrophy
Heroin toxicity	Hyperacute “spinal shock: Occurs after re-introduction of heroin after period of abstinence	Holocord involvement Long lesion
**Vitamin deficiencies**		
B12 deficiency (subacute combined degeneration)	Gastric bypass surgery Known vitamin deficiency Inflammatory bowel disease Megaloblastic anemia Peripheral neuropathy Often a chronic onset	Long segment Dorsal and/or lateral T2 hyperintensity, inverted “V” sign (due to bilateral dorsal column hyperintensity)
Folate deficiency	Use of dihydrofolate reductase inhibitors (e.g., methotrexate) Dietary limitations Malabsorption syndrome	Dorsal and/or lateral T2 hyperintensity (same as subacute combined degeneration)
Copper deficiency	Bariatric surgery Coexisting neuropathy Excess zinc ingestion (e.g., use of denture cream)	Dorsal and/or lateral T2 hyperintensity (same as subacute combined degeneration)
Vitamin E deficiency	Spinocerebellar syndrome Malabsorption syndrome (e.g., cystic fibrosis, resection of small intestine)	Often normal spinal MRI
**Malignancies**		
CNS lymphoma	Immunocompromised Multifocal presentation Progressive myelopathy and radiculopathy	Hyperintense on diffusion weighted imaging Poorly defined lesion with homogenous enhancement
Ependymoma	Low back pain Chronic presentation, leg weakness	Conus involvement Symmetric cord expansion Well demarcated Central pattern
Astrocytoma	Brown Sequard Syndrome Local pain Chronic presentation	Peripheral lesion Majority have patchy enhancement Poorly defined margins

#### Inflammatory disorders

Neurosarcoidosis can often present as LETM typically with subpial and/or ventral subpial enhancement, often at regions of disc herniations or spondylosis. A study of 62 sarcoidosis-associated myelopathy patients found discrete patterns on spinal MRI including LETM (45%), short tumefactive myelitis (23%), spinal meningitis/meningoradiculitis (23%), and anterior myelitis associated with areas of disc degeneration (10%) (57). The combination of central canal and dorsal subpial enhancement has been described in several cases of neurosarcoidosis and termed the “trident sign” (57, 58).

Systemic autoimmune disease may also be associated with myelitis, often with associated clinical features such as rash, sicca symptoms, Raynaud phenomenon, and abnormal serology in support of these rheumatological diagnoses.

#### Structural and vascular lesions

Compressive myelopathy such as cervical spondylosis may demonstrate gadolinium enhancement patterns that gradually resolve post-operatively. Ischemia due to dynamic compression may be seen as well (as in Hirayama disease due to anterior displacement of the dura during neck flexion). Hyperacute or acute onset is suggestive of a vascular myelopathy such as from primary spinal cord infarction, commonly seen as a complication from aortic surgery, although local thromboses and thromboemboli may cause this as well. Fibrocartilaginous embolism may also lead to ischemia which can occur following trauma such as lifting, exertion, or minor trauma from exercise. Ischemic myelopathies classically present with “owl eye” appearance which involves anterior horn gray matter in the vascular distribution of anterior spinal artery which is a vulnerable watershed region (55).

Dural arteriovenous fistulas are common mimics of immune-mediated myelopathies and present with stepwise worsening and fluctuating symptoms with Valsalva or exercise, which increases venous pressure, causing transient worsening of symptoms. This commonly involves the lower thoracic spinal cord and/or the conus. Patchy gadolinium enhancement may be seen as well as dilated peri-medullary veins that appear as flow voids on T2 weight sequences.

#### Neoplasms

Intramedullary tumors such as lymphomas are mimickers of immune-mediated myelopathies (56). Lymphomas may briskly respond to corticosteroids causing diagnostic confusion. Malignant lesions can become expansile over time and are often multifocal with additional cord or brain lesions. Metastatic lesions to the spinal cord may cause extrinsic compression or intramedullary metastases. Myelopathies may be a result of radiation or chemotherapy toxicity. Persistent gadolinium enhancement may be seen.

#### Toxic and/or metabolic etiologies

Subacute combined degeneration of the spinal cord is associated with B12 deficiency which is tract specific (dorsal and lateral columns) and may mimic paraneoplastic myelopathies. Other metabolic causes include vitamin E deficiency, copper deficiency, zinc toxicity (*via* inhibition of copper absorption) (54, 59). Heroin myelopathy has been reported after re-exposure to heroin after a period of abstinence, and may present acutely as spinal shock (60).

#### Idiopathic

Idiopathic transverse myelitis is a diagnosis of exclusion and should only be considered after exclusion of other diagnoses (1, 52). In some myelopathies, specific diagnosis may remain uncertain, and biopsy may be considered in select cases. Given the high potential morbidity of spinal cord biopsy, this is typically reserved for cases when management would be significantly altered such as with suspicion for spinal cord tumor for example. Importantly, in one series from Mayo Clinic diagnostic yield was low at 26%, suggesting biopsy be done only after rigorous alternative investigation (1).

### Treatment approaches

It is important to consider the pathogenic mechanism when selecting the treatment that is most likely to be beneficial in each condition. For each antibody syndrome, the treatment approach may differ but the generally include similar acute therapies including high dose corticosteroids with or without plasma exchange (PLEX) or intravenous immunoglobulin (IVIg). In those who have a paraneoplastic myelopathy, treatment of the underlying cancer portends a better prognosis than with immunotherapy alone. Rigorous evaluation to discover a malignancy is important, particularly in the antibodies known to be highly associated with malignancy. This should include physical examination, a mammogram in women and body computed tomography (CT). Body positron emission tomography (PET) improves sensitivity to evaluate cancer risk in those with negative CT scans. In PNS, treatment of the underlying malignancy is critical, and immunotherapy may be considered in addition to anti-neoplastic treatment. For most PNS, the associated antibody is commonly an intracellular target and a marker of a cytotoxic T cell mediated pathway as opposed directly pathogenic. Therefore, immunotherapies with a more direct action on depleting T cells (such as cyclophosphamide) are often preferred over antibody-targeted therapies like anti-CD20 agents. For some antibodies against intracellular targets (e.g., anti-Hu), immunotherapy may have limited success, while other intracellular antibodies (especially those that are not “classically paraneoplastic” such as anti-GFAP) may be more immunotherapy responsive.

For AQP4-IgG positive NMOSD, B-cell depleting agents such as rituximab has been used traditionally as standard of care with success (1). Since 2019 there are three FDA-approved therapies including eculizumab (61), satralizumab (62), and inebilizumab (62). For other antibody-mediated myelopathies, there are no randomized clinical trials that exist. Therefore, the current treatment approach tends to be highly variable and frequently includes steroid-sparing agents such as rituximab, chronic IVIg, mycophenolate, azathioprine, tocilizumab, or cyclophosphamide. Similarly, these have been utilized in MOGAD as well (63), with retrospective data has suggested IVIg appears to be a useful maintenance therapy in MOGAD to decrease frequency of demyelinating attacks (64).

## Conclusion

Myelopathies have a broad differential diagnosis with autoimmune and paraneoplastic etiologies being increasingly recognized. This includes well-described syndromes such as NMOSD and MOGAD, as well as less common antibody-associated disorders. Prompt recognition of these disorders is critical, as early, and aggressive immunotherapy provides the most optimal patient outcomes.

## Author Contributions

All authors listed have made a substantial, direct, and intellectual contribution to the work and approved it for publication.

## Funding

This work was supported by the Rocky Mountain MS Center and the Drake family in the name of Susan Drake.

## Conflict of interest

Author AP reports grants from University of Colorado and Rocky Mountain MS Center, consulting fees from Genentech/Roche and Alexion, and also receive honorarium from Medlink and publication royalties from Springer as co-editor of a textbook. The remaining authors declare that the research was conducted in the absence of any commercial or financial relationships that could be construed as a potential conflict of interest.

## Publisher's note

All claims expressed in this article are solely those of the authors and do not necessarily represent those of their affiliated organizations, or those of the publisher, the editors and the reviewers. Any product that may be evaluated in this article, or claim that may be made by its manufacturer, is not guaranteed or endorsed by the publisher.
